# Evaluation of factors associated with bulk milk somatic cell count and total plate count in Indonesian smallholder dairy farms

**DOI:** 10.3389/fvets.2023.1280264

**Published:** 2023-11-28

**Authors:** Achmad Fadillah, Bart H. P. van den Borne, Okti Nadia Poetri, Henk Hogeveen, Thomas Slijper, Herwin Pisestyani, Ynte H. Schukken

**Affiliations:** ^1^Business Economics Group, Wageningen University and Research, Wageningen, Netherlands; ^2^School of Business, IPB University, Bogor, Indonesia; ^3^Quantitative Veterinary Epidemiology, Wageningen University and Research, Wageningen, Netherlands; ^4^Department of Animal Diseases and Veterinary Public Health, School of Veterinary Medicine and Biomedical Science, IPB University, Bogor, Indonesia; ^5^Department of Economics, Swedish University of Agricultural Sciences (SLU), Uppsala, Sweden; ^6^Royal GD, Deventer, Netherlands

**Keywords:** dairy cattle, udder health, milk quality, total plate count, somatic cell count, generalized estimating equations

## Abstract

Increasing milk quality in smallholder dairy farms will result in a greater quantity of milk being delivered to milk collection centers, an increased milk price for farmers and consequently an improved farmers’ livelihood. However, little research on milk quality has been performed on smallholder farms in Southeast Asia. The objective of this study was to identify risk factors associated with somatic cell count (SCC) and total plate count (TPC) in Indonesian smallholder dairy farms. One dairy cooperative in West Java, Indonesia was selected based on its willingness to participate. All 119 member farmers in the cooperative, clustered in six groups, were interviewed and a bulk milk sample from all farms was collected in April 2022. Risk factors associated with dairy farms’ SCC and TPC were investigated using multivariable population-averaged generalized estimating equations (GEE) models. The mean geometric SCC and TPC from these farms were 529,665 cells/mL of milk and 474,492 cfu/mL of milk, respectively. Five risk factors including manure removal frequency, receiving mastitis treatment training, washing the udder using soap, number of workers, and ownership of the pasture area were associated with SCC. Two risk factors, manure removal frequency and dairy income contribution, were associated with TPC. These findings can therefore be used as a starting point to improve udder health and milk quality in Indonesia and other countries where smallholder farmers play a significant role in milk production.

## 1 Introduction

The dairy sector plays an important role in the economic development of several Southeast Asian nations, such as Indonesia, Malaysia, Vietnam, and Thailand (1). It provides income and employment opportunities for farmers and contributes to food security and nutrition (1–4). The vast majority of milk in Southeast Asia is produced on smallholder dairy farms. In recent years, the sector has experienced substantial growth due to population expansion, increasing *per capita* income, the rise of the middle class, and growing awareness of the health benefits of milk and dairy products (5, 6).

Consumption of milk *per capita* in Southeast Asian countries was projected to increase by 3% annually between 1997 and 2020 (7, 8). However, microbial contamination remains a significant concern for milk spoilage, food safety and can pose health risks to consumers (9–12). Dealing with these risks requires monitoring and managing milk quality parameters in dairy farms to ensure milk quality and food safety.

The primary indicators used to measure the quality and hygiene of bulk milk are somatic cell count (SCC) and total plate count (TPC; 13, 14). Monitoring SCC values provides an understanding of milk quality, udder health status, and the presence of subclinical mastitis (15, 16). On the other hand, TPC values in milk indicate predominantly bacterial contamination resulting from dairy farm practices such as milking, handling, and milk storage (17, 18).

Despite its importance in world-wide milk production, the quality of milk produced by smallholder dairy farmers is often suboptimal due to poor cleaning and disinfection practices, the suboptimal storage of milk, inadequate sanitation and poor hygiene in the milking environment (5, 19, 20). Elevated levels of SCC and bacterial contaminants in milk can have adverse effects on its quality and safety, leading to reduced milk yield and decreased shelf-life of milk and its derived products (21, 22). Moreover, high SCC and TPC levels can result in the rejection of milk-by-milk collection centers and processors because it fails to meet the required milk quality standards, resulting in substantial economic losses for dairy farmers (23, 24).

Creating knowledge on SCC and TPC risk factors is crucial in assisting farmers to decrease SCC and TPC to meet milk quality standards. Numerous risk factor studies have been conducted worldwide, with a significant emphasis on regions such as Europe and North America (25–28). Findings of such studies have contributed substantially to the realization of the National Mastitis Council’s ten-point mastitis control program, shedding light on critical risk factors and effective management practices. However, such knowledge is limited available for smallholder dairy systems in Southeast Asia (29–31).

The aim of this paper is therefore to determine milk quality and identify risk factors associated with SCC and TPC levels in bulk tank milk of smallholder dairy farms in Indonesia. The study sheds light on the challenges faced by smallholder dairy farmers in improving milk quality.

## 2 Materials and methods

### 2.1 Study design, area, and population

**Figure 1:**
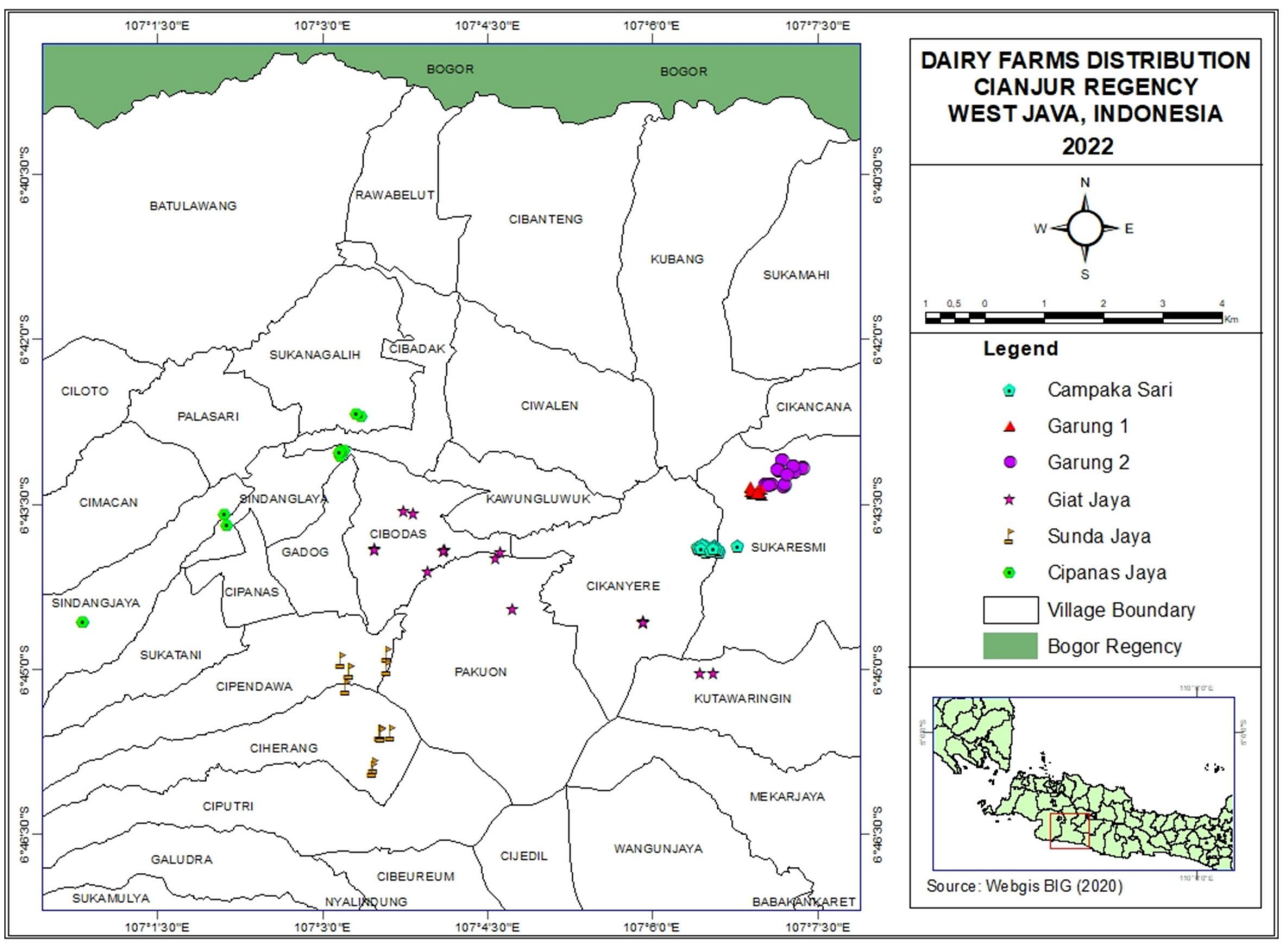
Geographical location of the 119 smallholder dairy farms included in the survey. Different colors and symbols indicate the six farmers’ groups.

A cross-sectional study among smallholder dairy farmers in Cianjur District, West Java, Indonesia was conducted in April 2022. In Indonesia, dairy cooperatives are formal collective organizations formed by local dairy farmers, serving pivotal roles such as operating milk collection centers, providing market access, facilitating input supply, offering training, extension services, and animal health services, and advocating for their members’ interests at the local, regional, and national level. Relevant stakeholders, experts, and representatives from several dairy cooperatives were consulted to select one dairy cooperative from several potential candidates in West Java, Indonesia. After careful consideration of factors such as willingness to collaborate, a well-established reputation, convenient proximity to the laboratory facility in Bogor, and the size of the cooperative which allows for comprehensive farm visits, and the alignment of its activities with the current study, KPS Cianjur Utara cooperative was chosen. In the chosen cooperative, the payment scheme for milk currently does not incorporate SCC and TPC values. All of its 119 milk producing member farms were included in the study. The geographical distribution of the dairy farmers included in the study is displayed in Figure 1 and indicates the clustering of farmers. These clusters were used to form six geographically connected farmers groups. In Cianjur District, farmers’ groups play an important role in facilitating assistance and extension programs by the cooperatives and the local government. Farmers’ groups, in collaboration with the cooperative, also provide services to farmers (e.g., the facilitation of a milk collection point, veterinary services) and collectively provide and distribute feed and dairy farm inputs among their members.

### 2.2 Method of data collection

Ethical approval for this study was obtained from the Social Sciences Ethics Committee at Wageningen University and Research (WUR), Netherlands.

Farmers were interviewed using a paper-based questionnaire which was translated to and paraphrased in Bahasa Indonesia from English. Before administration, the questionnaire was pre-tested on 6 farmers and adapted according to their feedback. The data was collected by a trained team of 3 enumerators and 3 paramedics of the cooperative that visited the selected farms. Enumerators are individuals tasked with data collection from smallholder dairy farms through surveys and interviews. For this study, we involved 3 enumerators, all of whom possessed university degrees—2 in veterinary science and 1 in social science. Paramedics, on the other hand, are healthcare staff hired by the dairy cooperative to provide animal health services to dairy cattle and offer assistance to smallholder farmers who are cooperative members, particularly in matters related to dairy cattle health and management. To support our survey efforts, animal data recording, and milk sampling, we involved 3 paramedics from the selected dairy cooperative. Enumerators and paramedics were fluent in Bahasa Indonesia and the local language, Sundanese. To improve the quality of data collection, each filled questionnaire was immediately checked by a research data collection supervisor. If there were missing or illogical data, confirmation was sought with the dairy farmers, paramedics, or cooperative staff. The participating farmers were compensated to cover the opportunity cost of their time spent during interviews, as well as to enhance participation rates and ensure data accuracy.

### 2.3 Survey data

Information on 55 variables putatively associated with farmers’ milk quality parameters (SCC and TPC) was collected during the farm visits. It included data on socio-demographics, farm characteristics, dairy farm management practices, as well as risk factors regarding milk quality. The selection of these variables was based on a literature review of factors affecting milk quality in smallholder settings and the experience of the current research team with milk quality programs and udder health. The variables for this study were divided into 5 categories:

1. *socio-demographics* (age, gender, education, number of family members, main occupation, second occupation, dairy income contribution, and dairy business experience),
2. *farm characteristics* (livestock unit, barn size, barn ownership, land area for growing grass, pasture area ownership, total labor, family labor, paid labor, village, subdistrict, farmers’ group identification, milk production, water source, and type of bedding),
3. *animal health services and training frequency* (paramedic visits, frequency of animal health trainings, udder health trainings, and mastitis treatment trainings),
4. *dairy management practices* (cleaning stall, frequency of manure removal, using and changing of bedding, number of mastitis treatment days, visually checking the udder before milking, testing of new cows with the Californian mastitis test, pre-stripping, checking temperature and swelling of the udder before milking, checking the willingness of the cow for eating and drinking, being aware of subclinical mastitis, treating mastitis cows by themselves, using antibiotics at dry off, using a towel for cleaning the udder, post milking teat disinfection, milking mastitis cows at the end, isolating mastitis cows, washing of cows, washing the udder using soap, cleaning towel, number of cows per towel, washing hands, cleaning of milking equipment, milking methods, and level of feeding),
5. *animal disease or mastitis impact* (reduction of milk yield, number of days with reduced milk yield, return to pre-treatment milk yield levels after cure, and mastitis production impact).

### 2.4 Milk sampling and laboratory analyses

After milking their cows, farmers bring the milk to a milk collection center, where the sample was taken. In the milk collecting centers, 50 mL of bulk milk from each farm was stored in labeled tubes. Tubes were then placed on ice in a cooling box and delivered to the laboratory on the same day to prevent milk spoilage and the growth of bacteria.

Bulk milk samples were tested in the Laboratory of Veterinary Public Health at the School of Veterinary Medicine and Biomedical Science of IPB University (Indonesia). Samples were analyzed for somatic cell count (SCC), total plate count (TPC) and six other milk composition parameters including fat, solids nonfat, total solids, lactose, and protein content and milk density.

#### 2.4.1 Somatic cell count

SCC was determined using the Breed method (32, 33). Briefly, after homogenization, 0.01 mL of milk was pipetted on an object glass using a Breed pipette and was spread to form a 1 cm^2^ square using an elbow-tipped loop. The object glass was fixed with a Bunsen flame after which a Breed staining was performed. The object glass was first immersed in an ether alcohol solution for 2 min, then in Löffler’s methylene blue solution for 1–2 min, and lastly in 96% ethanol for 1 min to remove the remaining attached dyes. After drying, somatic cells were counted using a light microscope. The number of somatic cells was counted using 30 viewpoints, summed and divided by the number of viewpoints to determine the average number of somatic cells for the sample. The final SCC was determined by multiplying the average number of somatic cells with the microscopy factor (400,000).

#### 2.4.2 Total plate count

TPC was determined using the pour plate method (34). A total of 1 mL milk was added to a solution of 9 mL 0.1% buffer peptone water (BPW) to obtain a 10^−1^ dilution. After homogenization, serial dilutions of 10^−2^, 10^−3^, 10^−4^, 10^−5^, and 10^−6^ were prepared to select 3 consecutive dilutions for cultured. A total of 1 mL of milk sample from 3 selected dilutions was transferred into a petri dish after which 10 mL to 15 mL of plate count agar was poured. After homogenization and solidification, the agar culture was incubated at 35°C for 24 to 48 h in an inverted position. The calculation was carried out for all microorganisms (both large and small) that grew in a petri dish. Petri dishes containing 25 to 250 colonies were recorded along with the number of dilutions made. Colony counts were determined according to the rules of the American Public Health Association (APHA):

$\begin{array}{l} \mathrm{Number}\;\mathrm{of}\;\mathrm{microorganisms}\;\left(\mathrm{cfu}/\mathrm{ml}\right)=\mathrm{Number}\;\mathrm{of}\;\mathrm{colonies}\times \\ 1/\mathrm{dilution}\;\mathrm{factor} \end{array}$

#### 2.4.3 Milk composition

Fat, solids nonfat, lactose, and protein content and milk density were determined using the Lactoscan SP milk analyzer (Milkotronic LTD., Nova Zagora, Bulgaria) following the guidelines of the manufacturer. The total solid parameter was accumulated from the fat and solid-non-fat components.

### 2.5 Data management and statistical analysis

Data management and statistical analysis were conducted using Stata/SE version 17.0 (StataCorp LLC, Texas, United States). The quality and composition (SCC, TPC, fat, solids nonfat, total solids, lactose, and protein content and milk density) of the bulk milk sample, overall and within each farmers’ group, was determined using descriptive statistics. The median differences of fat, solids nonfat, total solids, lactose, and protein content and milk density among farmers’ group were tested using Kruskal-Wallis’s equality of populations rank test.

To identify risk factors associated with milk quality, SCC and TPC were in focus. Given their skewed distributions, the natural logarithm of SCC (LnSCC) and TPC (LnTPC) was determined. Factors associated with LnSCC and LnTPC were separately analyzed using multivariable population-averaged generalized estimating equations (GEE) models in complete-case analyses. Initial data exploration using null mixed-effects models identified that there was substantial clustering of farms with farmers’ groups since their intraclass correlation coefficients were 25.9 and 13.3% for LnSCC and LnTPC, respectively. To correct for this clustering of farms within farmers’ groups in relation to dairy management practices, animal health services, and geographic location, farmers’ group identification was the clustering variable in the GEE models. The model further incorporated the exchangeable correlation structure (35), assuming an equable level of correlation across farms. In the model, the outcome variables, LnSCC and LnTPC, were continuous variables, normally distributed, and analyzed using the identity link function. The GEE model was defined as follows:

Y=β0+βi∗variables+ε

where *Y* represents the LnSCC and LnTPC, *β_0_* denotes the intercept, *βi* represents the regression coefficients for the variables, and ε signifies the error term.

Linear relationships between the continuous variables and both outcome variables were checked using scatterplots based on 10-percentile data. Variable selection started with univariable regression models in which 55 variables were individually tested for their association with LnSCC or LnTPC. Variables that had a *p*-value below 0.20 in the Type III test were selected for further analysis. Correlation among pairs of selected explanatory variables was assessed thereafter to avoid multicollinearity. The Cramers’ V correlation test was used to determine the correlation between two categorical variables while the Spearman’s correlation test was used to investigate correlations between two numerical variables with nonlinear data and correlations between one categorical variable with one numerical variable. If two variables had a correlation coefficient higher than 0.5, one of the variables was selected in our analysis. This included the frequency of farmers’ training on topics of animal health and udder health. These two variables had a strong correlation with the frequency of training on mastitis treatment (Cramers’s V = 0.8 and 0.9, respectively) in the analysis for LnSCC. The latter was selected for the multivariable analysis as it represented training in mastitis treatment. Dairy income contribution and second occupation were also strongly correlated (Cramers’s V = 0.8) of which dairy income contribution was included in the analysis for LnTPC. Thereafter, a backward selection process was used for model specification until all selected variables significantly contributed to the model (*p* < 0.5), based on the Type III test, or were considered confounders. The latter was defined when effect estimates changed more than 25% when removing a variable from the model. Interaction terms were not evaluated. The quasi-likelihood under the independence model criterion (QIC) was employed as a goodness-of-fit measure to evaluate the final GEE model (36). A post-hoc power analysis was performed using the observed mean and standard deviation values for LnSCC and LnTPC. Statistical significance was defined at *p* < 0.05 and the observable difference in outcome values was calculated at a power of 80%.

## 3 Results

### 3.1 Description of study population and milk quality

The general demographic and farm characteristics of the 119 participating farms are provided in Table 1. Dairy farming in Cianjur, West Java, Indonesia is dominated by smallholders who have an average livestock unit of 5.44 and dairy labor of 2 persons per farm. The average age, education, and dairy business experience of dairy farmers were 44 years, 8 years, and 16 years, respectively.

**Table 1:** Characteristics of 119 smallholder dairy farms located in Cianjur District, West, Java, Indonesia that were included in the survey.

Category	Variable	Mean	STD	5% percentile	95% percentile
Farmer characteristics	Age (years)	43.47	11.65	22	62
	Education (years)	7.45	3.01	6	15
	Dairy business experience (years)	15.82	9.18	4	35
Farm characteristics	Livestock units	5.44	3.50	1.40	11.80
	Dairy labor (persons)	1.94	0.76	1	3

The average value of milk quality parameters was 3.04% (SD = 0.99), 5.72% (SD = 1.05), 8.76% (SD = 1.66), 3.14% (SD = 0.57), 2.09% (SD = 0.38), and 1.02 gr/cm^3^ (SD = 0.00), for fat, solids nonfat, total solids, lactose, and protein content and milk density, respectively. There were no significant differences in the median of the six milk quality parameters among farmers’ groups.

The descriptive statistics of the LnSCC and LnTPC per farmers’ group are presented in Table 2. The mean overall LnSCC was 13.18, which corresponds with a geometric SCC of 529,665 cells/ml. Giat Jaya and Garung 2 had the highest and lowest average LnSCC with levels of 14.12 and 12.59, respectively. The mean overall LnTPC was 13.07, which is a geometric TPC of 474,492 cfu/mL. Garung 2 and Sunda Jaya had the highest and the lowest LnTPC levels with 14.47 and 12.10, respectively. Post-hoc power calculation indicated that with a power of 80% and a significance value of 5%, a difference of 0.5 LnSCC and 1.0 LnTPC units would be observable.

**Table 2:** Distribution of the natural logarithm of SCC (LnSCC) in cells/mL and the natural logarithm of TPC (LnTPC) in cfu/mL per farmers’ group.

Farmers’ group	*N*	LnSCC				LnTPC			
		Mean	STD	5% percentile	95% percentile	Mean	STD	5% percentile	95% percentile
Campaka Sari	32	13.23	0.86	11.51	14.38	12.54	2.10	9.68	16.30
Garung 1	20	12.66	0.88	11.00	13.67	13.42	1.80	11.05	16.19
Garung 2	26	12.59	0.90	11.00	14.95	14.47	1.96	11.51	17.21
Giat Jaya	17	14.12	1.12	12.30	16.44	12.84	2.46	9.99	17.28
Sunda Jaya	13	13.60	0.89	12.30	15.14	12.10	1.92	9.85	15.64
Cipanas Jaya	11	13.47	0.94	11.98	15.27	12.20	1.56	10.55	16.30
Overall	119	13.18	1.05	11.29	15.14	13.07	2.15	10.04	17.06

### 3.2 Factors associated with LnSCC

The results of the GEE models for LnSCC are presented in Table 3. Five variables were significantly associated with LnSCC in the model considering all variables. Farmers who removed manure 3 times per day had a LnSCC that was 0.78 (95% CI: −1.11 to −0.44) units lower compared to those who removed manure 1 to 2 times per day. Farmers who received one mastitis treatment training in the last 12 months had 0.71 units lower LnSCC (95% CI: −1.14 to −0.28) compared to farmers that did not receive any mastitis treatment training. Those who received 3 to 6 mastitis treatment training had a LnSCC that was 0.77 units lower (95% CI: −1.20 to −0.33). Farmers who wash the udder of their cows after milking using soap had a LnSCC that was 1.73 (95% CI: −3.34 to −0.13) units lower compared to those who do not use soap. Furthermore, farmers who have 3–6 workers had a LnSCC that was 0.47 (95% CI: 0.00 to 0.94) units higher compared to those who have only 1 worker. Finally, ownership of the pasture area was also associated with LnSCC. Its value was 0.98 (95% CI: −1.74 to −0.21) units lower when farmers borrowed land for growing grass and 1.08 (95% CI: −1.75 to −0.42) units lower when they used communal or public land for growing their grass, in comparison to farmers who do not own any land for growing grass.

**Table 3:** Final generalized estimating equations models explaining the natural logarithm of somatic cell count (SCC) of 119 smallholder dairy farms in Cianjur District, West Java, Indonesia.

Factor and category	*N*	Coef.^a^	SE	Wald *p*-value	Overall *p*-value^b^
Intercept		14.68	0.35		
Manure removal frequency					<0.001
≤ 2 times/day	71	Ref.			
3 times/day	39	−0.78	0.17	<0.001	
≥ 4 times/day	9	−0.40	0.29	0.176	
Mastitis treatment trainings per year					0.001
No	30	Ref.			
1 training	32	−0.71	0.22	0.001	
2 trainings	30	−0.25	0.21	0.228	
≥ 3 trainings	27	−0.77	0.22	0.001	
Washing udder using soap					0.031
Not	100	Ref.			
Before milking	15	−0.45	0.24	0.063	
After milking	1	−1.73	0.82	0.034	
Before & after milking	3	0.45	0.47	0.339	
Total labor					0.046
1 person	33	Ref.			
2 persons	64	−0.03	0.18	0.891	
≥ 3 persons	22	0.47	0.24	0.048	
Pasture area ownership					0.012
Do not own any pasture area	7	Ref.			
Owned	31	−0.65	0.34	0.058	
Rented	36	−0.65	0.34	0.059	
Borrowed	12	−0.98	0.39	0.012	
Communal/ public land	33	−1.08	0.34	0.001	

a The QIC value was 110.88.

b *p*-value based on the type III test.

### 3.3 Factors associated with LnTPC

Two factors were associated with dairy farmers’ LnTPC in the final GEE model as shown in Table 4. First, farmers who removed manure 4–6 times per day had LnTPC levels that were 1.46 (95% CI: 0.07 to 2.84) units higher compared to farmers who removed manure 1–2 times per day. Second, herds in which the dairy income contributed 50 to 75% to their total income were associated with LnTPC levels that were 1.01 (95% CI: 0.22 to 1.80) units higher compared to those herds where the dairy income contributed more than 75% of the total income.

**Table 4:** Final generalized estimating equations models explaining the natural logarithm of total plate count (TPC) of 119 smallholder dairy farms in Cianjur District, West Java, Indonesia.

Factor and category	*N*	Coef.^a^	SE	Wald *p*-value	Overall *p*-value^b^
Intercept		12.25	0.40		
Manure removal frequency					0.044
≤ 2 times/day	71	Ref.			
3 times/day	39	0.79	0.42	0.062	
≥ 4 times/day	9	1.46	0.71	0.039	
Dairy income contribution					0.030
>75%	79	Ref.			
>50%–75%	34	1.01	0.40	0.012	
>25%–50%	6	0.96	0.82	0.239	

a The QIC value was 505.54.

b *p*-value based on the type III test.

## 4 Discussion

This study assessed the SCC and TPC levels, as well as identified the factors associated with these milk parameters in the context of smallholder dairy farming in the Cianjur District of West Java, Indonesia. In this regard, we examined the geometric mean SCC of the Cianjur dairy farmers in our study, which was found to be 529,665 cells/ml. This finding emphasizes the importance of enhancing udder health management practices to align SCC levels with acceptable standards, specifically adhering to the maximum limit of 400,000 cells/ml of milk stipulated in the Indonesian National Standard (No. SNI 3141.1:2011) for fresh milk (37). A high SCC is not only a milk quality problem, but high SCC values also indicate udder health problems (15, 38). In general, a threshold SCC value of 200,000 cells/ml of milk is used to identify a cow with subclinical mastitis (16, 39–45). A high bulk milk SCC indicates towards a high prevalence of subclinical mastitis leading to reduced productivity, profitability and animal welfare. The geometric mean Total Plate Count (TPC) among Cianjur dairy farmers was found to be 480,000 cfu/mL, which falls below the maximum permissible limit of 1,000,000 cfu/mL of milk as set by the Indonesian National Standard (No. SNI 3141.1:2011) for fresh milk (37). However, there is still room for improvement given the variation seen between farms. Also, it cannot be ruled out that TPC and other milk quality parameters levels may change over time given the cross-sectional design of our study. Currently, TPC is not a factor considered by Cianjur dairy cooperative in determining the milk price for farmers. Introducing payment schemes that incentivize smallholder farmers to increase the hygienic quality of milk potentially improves the overall quality of milk in the supply chain (46, 47). Additionally, no statistically significant differences were observed in the median values of the six milk quality parameters (fat, solids nonfat, total solids, lactose, protein content, and milk density) among the farmers’ groups.

As expected, our study found that management practices, such as manure removal frequency and udder washing with soap were significantly associated with SCC levels. Accumulation of manure in the barn can create a favorable environment for mastitis pathogens, which can cause subclinical mastitis (48–50). Hence, frequent removal of manure is critical for improving milk quality and reducing SCC levels (19, 51–53). However, we found that frequent removal of manure was associated with increased levels of TPC. This could be attributed to the practice used by smallholder farmers in West Java, Indonesia to remove manure in the barn by hosing of water (wet cleaning). Such wet conditions are conducive to bacterial growth and the spread of bacteria around the barn, cows, and milk (54, 55). These findings suggest that there may be a need for alternative practices of manure removal (such as dry cleaning) to minimize the risk of bacterial contamination in the barn, cows, and milk.

Pre-milking and post-milking procedures are critical in minimizing bacterial contaminants on the cow’s udder and teats. Washing the udder with soap before milking and post milking teat disinfection are effective practices in reducing bacterial contamination. Proper washing and drying of the udder and teat are also important in maintaining udder health, reducing mastitis risk, and minimizing SCC levels in milk (56, 57).

Effective mastitis control and treatment are crucial for maintaining milk quality and preventing economic losses for smallholder dairy farmers. Several studies have shown that mastitis control and treatment training can improve the knowledge and practices of farmers in preventing and managing intramammary infections (25, 58). Enhancing milk quality and effectively managing mastitis begins by ensuring that farmers possess a thorough awareness of the milk quality parameters (59). In Cianjur, dairy farmers receive mastitis control and treatment training from various organizations, including dairy cooperatives, the local government, dairy companies, and universities collaborating with farmers’ groups. Dairy cooperatives and farmer groups in Indonesia play complementary roles in supporting dairy farming. Cooperatives are more formalized entities, primarily focused on marketing, processing, and providing various services to their members. In contrast, farmer groups are informal associations that facilitate collaborative farming, knowledge sharing, and community support among individual farmers. The training covers various topics such as milking hygiene, proper milking procedures, early detection of mastitis infections, udder health management, and appropriate treatment of infected cows, which can improve dairy farmers’ knowledge and practices in preventing and managing mastitis infections (60).

The study identified some factors associated with SCC. First, a positive association between total labor and increasing SCC in dairy farms was found. This is in line with previous studies that found that hiring more labor on farms was associated with increasing SCC levels (61, 62). The lack of standardized practices and variation in routines among hired labor highlight the need for implementing standard operating procedures on the farm. Second, our research revealed that farmers who lack land for growing grass had higher levels of SCC. As an alternative, farmers frequently used vegetable waste sourced from traditional markets as a substitute for feeding their dairy cows. However, heavy metal contamination, such as lead (Pb), copper (Cu), and mercury (Hg), has been found in traditional market vegetable waste in Yogyakarta, Indonesia (63). It is therefore important to properly process and check vegetable waste for physical contamination before being fed to the cows.

Predictably, better general hygiene practices were associated with a lower number of bacteria in bulk tank milk. This study found that farmers who used and/or changed their bedding regularly had lower total plate counts in their milk samples compared to farmers who did not use bedding in their barns. This finding is consistent with previous studies that have shown the importance of managing bedding to control bacterial growth and udder infections (64–68).

Smallholder farmers who had a higher dairy income contribution had a lower level of TPC in their bulk tank milk, indicating that they may have better knowledge of hygiene practices. This suggests that improving the knowledge of smallholder farmers regarding milk quality and hygiene practices can lead to greater implementation of these practices, resulting in an improved milk quality (20). Additionally, smallholder farmers who implement good hygiene practices may produce higher quality milk, which in turn may contribute to higher revenues or milk prices. The implementation of an incentive based on milk quality by the cooperative could serve as an additional motivating factor for farmers to adopt and maintain these essential hygiene practices.

Due to our cross-sectional study design, we are unable to prove causal effects and caution is required when interpreting our results. Rather, our results should be interpreted as associations. Another limitation pertains to the relatively modest sample size of dairy farms present in the cooperative that we studied. However, census data from one dairy cooperative was available and post-hoc power calculations indicated that an observed difference in 0.5 LnSCC units and 1.0 LnTPC units would be detectable. This was also the approximate size of effects present in the final models (Tables 3, 4). Furthermore, we applied the GEE population-averaged model in the analysis to provide robust and reliable inferences about associations between explanatory variables and the outcome variables at the population level (69–71). Therefore, this study’s findings can be used to make inferences about the wider population of smallholder dairy farms in tropical Southeast Asian countries, in which dairy farms have similar characteristics as in the study area.

## 5 Conclusion

This study identified milk quality parameters and examined the various risk factors that were associated with levels of bulk milk somatic cell count (SCC) and total plate count (TPC) smallholder dairy farms in Indonesia. To achieve lower levels of SCC and TPC, several policy recommendations can be implemented. Firstly, it is crucial to encourage smallholder dairy farmers to adopt good hygiene practices, such as regular and thorough manure removal at least three times per day, proper udder washing using soap before and after milking, and the regular utilization and replacement of bedding in the barn. Secondly, it is recommended that farmers’ groups, cooperatives, and local government bodies collaborate to develop effective extension and training programs aimed at enhancing farmers’ knowledge and skills in mastitis management and milk quality. Lastly, cooperatives and dairy companies should establish a comprehensive system for monitoring milk quality parameters, including SCC and TPC, at milk collecting centers. This system can involve regular inspections, sample testing, and compliance checks to ensure consistent adherence to the recommended practices. Therefore, these findings and recommendations can also serve as an initial reference for enhancing udder health and milk quality in other countries where smallholder farmers play a crucial role in milk production. By implementing these measures, it is possible to significantly improve the overall quality of milk and udder health, benefiting both smallholder farmers and the dairy sector as a whole.

## Data availability statement

The raw data supporting the conclusions of this article will be made available by the authors, without undue reservation.

## Ethics statement

The studies involving humans were approved by The Social Sciences Ethics Committee at Wageningen University and Research (WUR), Netherlands. The studies were conducted in accordance with the local legislation and institutional requirements. The participants provided their written informed consent to participate in this study.

## Author contributions

AF: Conceptualization, Formal analysis, Methodology, Project administration, Visualization, Writing – original draft, Writing – review & editing. BB: Conceptualization, Formal analysis, Methodology, Writing – review & editing. OP: Methodology, Writing – review & editing. HH: Conceptualization, Methodology, Supervision, Writing – review & editing. TS: Project administration, Writing – review & editing. HP: Methodology, Writing – review & editing. YS: Conceptualization, Methodology, Supervision, Writing – review & editing.
